# Coffee Pulp from Azores: A Novel Phytochemical-Rich Food with Potential Anti-Diabetic Properties

**DOI:** 10.3390/foods14020306

**Published:** 2025-01-17

**Authors:** Anabela S. G. Costa, Juliana A. Barreto Peixoto, Susana Machado, Liliana Espírito Santo, Thiago F. Soares, Nelson Andrade, Rui Azevedo, Agostinho Almeida, Helena S. Costa, Maria Beatriz Prior Pinto Oliveira, Fátima Martel, Jesus Simal-Gandara, Rita C. Alves

**Affiliations:** 1 Network of Chemistry and Technology/Associated Laboratory for Green Chemistry (REQUIMTE/LAQV), Department of Chemical Sciences, Faculty of Pharmacy, University of Porto, 4050-313 Porto, Portugal; acosta@ff.up.pt (A.S.G.C.); jpeixoto@ff.up.pt (J.A.B.P.); smachado@ff.up.pt (S.M.); lsanto@ff.up.pt (L.E.S.); up201902664@edu.ff.up.pt (T.F.S.); nandrade@med.up.pt (N.A.); rmazevedo@ff.up.pt (R.A.); aalmeida@ff.up.pt (A.A.); helena.costa@insa.min-saude.pt (H.S.C.); beatoliv@ff.up.pt (M.B.P.P.O.); 2 Nutrition and Bromatology Group, Department of Analytical Chemistry and Food Science, Faculty of Science, University of Vigo, E-32004 Ourense, Spain; jsimal@uvigo.es; 3 Unit of Biochemistry, Department of Biomedicine, Faculty of Medicine of Porto, University of Porto, 4200-319 Porto, Portugal; fmartel@med.up.pt; 4 Department of Food and Nutrition, National Institute of Health Dr. Ricardo Jorge, I.P., Av. Padre Cruz, 1649-016 Lisbon, Portugal; 5 Instituto de Investigação e Inovação em Saúde (i3S), University of Porto, 4200-135 Porto, Portugal

**Keywords:** coffee by-product, valorization, sustainability, chemical composition, bioactivity, Caco-2 cells

## Abstract

Coffee pulp, a by-product of wet coffee processing, shows significant potential in the food and health domains, but its real applications remain underexplored. This work investigated the chemical composition and bioactive properties of coffee pulp from São Miguel Island (Azores, Portugal). The studied coffee pulp exhibited high fiber content (52% dw), mostly insoluble; notable mineral levels (10.6%), mainly K, Ca, and Mg; and 6% dw of total amino acids, with hydroxyproline, aspartic acid, glutamic acid, and leucine in higher amounts. Despite containing low fat (1.6% dw), mainly saturated, it also showed considerable amounts of polyunsaturated fatty acids with a favorable n6/n3 ratio (1.40) and vitamin E (α-, β-, and γ-tocopherols). Its antioxidant capacity can be partially explained by the chlorogenic acid content (9.2 mg/g dw), and caffeine (0.98%) was present in similar amounts to those observed in some arabica coffee beans. A decrease in glucose uptake in Caco-2 cells was found, but not in fructose, suggesting selective inhibition of SGLT1 and potential antidiabetic effects. These results show that Azorean coffee pulp has potential as a sustainable and bioactive ingredient for incorporation into functional foods or dietary supplements.

## 1. Introduction

A fresh coffee cherry contains approximately 35–45% of pulp [1,2]. With global coffee production reaching around ten million tons of raw beans annually [3], a substantial amount of this by-product is generated, underscoring the need for an effective means of valorization. Moreover, this by-product is frequently released into natural ecosystems, leading to harmful environmental effects due to the substantial accumulation of organic matter and phytotoxic compounds. However, coffee pulp holds significant potential as a functional ingredient for innovative applications in the food industry [1,2,4,5]. In fact, although the use of dried coffee pulp for food purposes is not usual in Portugal or other European countries, significant progress was made in 2022 when the European Union (EU) approved the commercialization of dried coffee pulp from *Coffea arabica* L. and *Coffea canephora* Pierre ex A. Froehner, along with its infusion, as a traditional food from third countries [6]. This decision was based on the long-standing history of safe consumption of coffee pulp and its beverage in countries such as Yemen, Ethiopia, and Bolivia, where they have been traditionally used [6]. As a result, the market for coffee pulp is expected to grow, highlighting the need for a comprehensive understanding of its potential health effects when consumed.

Overall, coffee pulp is recognized as a good source of antioxidants, particularly phenolics, such as chlorogenic acids, caffeic acid, ferulic acid, and coumaric acid [7]. Its chemical composition includes ~50% carbohydrates, 20% dietary fiber, 10% protein, 2.5% lipids, and 1.3% caffeine [4], making coffee pulp highly versatile. In addition to potential applications in bioethanol production [8] or as composting material [1,2], its anthocyanin content also makes it suitable as a natural food colorant [9]. It can also be processed into flour, containing 18% dietary fiber [4,10], or be used in functional infusions [1]. Coffee pulp can also undergo hydrolysis to recover sugars from the matrix, namely xylose, arabinose, fructose, glucose, sucrose, and maltose [4]. Several studies have also demonstrated a range of bioactive properties, including antioxidant, anti-inflammatory, hypolipidemic, hepatoprotective, antidiabetic, and antiobesity, as well as the capacity to modulate gut microbiota [11,12,13,14].

In this study, we conducted, for the first time, a comprehensive analysis of the chemical composition and bioactive potential of coffee cherry pulp from S. Miguel, Azores (Portugal), focusing on its antioxidant and antidiabetic properties. Although not yet well known in the international market or even among the general population, the Azores archipelago boasts a small but promising coffee production. These volcanic islands provide unique microclimatic conditions, such as mild temperatures (15 °C in winter to 27 °C in summer) and high humidity (76%), which contribute to optimal coffee plant growth [15]. These distinct environmental factors bring novelty and significance to this study, as they may influence the chemical and bioactive profiles of this by-product. To the best of our knowledge, this is the first detailed report on the nutritional profile of Azorean coffee pulp, covering its mineral composition, amino acid and fatty acid profiles, vitamin E content, free sugars, chlorogenic acids, and caffeine contents. Additionally, we explored its antioxidant and antidiabetic potential, including the first evaluation of sugar uptake inhibition by coffee pulp extracts in an intestinal cell line. Our findings can offer new insights into the unique qualities of Azorean coffee pulp and its potential applications.

## 2. Materials and Methods

### 2.1. Reagents, Standards and Materials

Kjeldahl tablets were purchased from Merck (Darmstadt, Germany). The dietary fiber assay kit and celite were provided by Sigma-Aldrich (St. Louis, MO, USA).

For mineral analysis, nitric acid (≥69% (*w*/*w*) Trace Metal™ was purchased from Fisher Scientific (Leicestershire, UK), hydrogen peroxide (30% *w*/*w*) Suprapur^®^ was provided by Merck (Darmstadt, Germany), and hydrochloric acid (>30% *w*/*w*) TraceSELECT™ was supplied by Honeywell Fluka™ (Seelze, Germany). Certified reference materials (BCR-679, ERM-BB422, ERM-BC382) were provided by the European Commission, Joint Research Centre (Brussels, Belgium). A 10 µg/mL internal standard (Mix1-SCP-IS7) and single-element stock solutions (1000 µg/mL) of gallium (Ga), rhodium (Rh), and calcium (Ca) were from SCP SCIENCE (Quebec, QC, Canada); single-element stock solutions (1000 µg/mL) of iron (Fe), magnesium (mg), sodium (Na), and potassium (K) for Atomic Absorption Spectroscopy were from Fluka (Seelze, Germany); Sigma-Aldrich (Buchs, Switzerland) provided phosphorus (P) and mercury (Hg) single-element standards (1000 µg/mL); and the ICP multi-element standard solution (21 elements) was obtained from Supelco (Darmstadt, Germany).

For chromatographic analyses, the following standards/reagents were used: standards of amino acids (98.0%–≥99.5%), tocopherols (α-, β, λ-, and δ-), fructose, glucose, and sucrose, as well as the Supelco 37 Component FAME Mix, were all provided by Sigma-Aldrich (St. Louis, MO, USA); Larodan (Solna, Sweden) provided tocotrienols (α-, β-, λ-, and δ-) and tocol; L-norvaline was purchased from Sigma (Deisenhofen, Germany); caffeine and chlorogenic acids (3-, 4-, and 5-caffeoylquinic acids) were supplied by Honeywell Riedel-de Haën^TM^ (Seetze, Germany). The derivatization reagents (9-fluorenylmethyl chloroformate and o-phthalaldehyde/3-mercaptopropionic acid) were purchased from Agilent Technologies (Palo Alto, CA, USA). For eluent preparation, 1,4-dioxane was obtained from Sigma-Aldrich (St. Louis, MO, USA); HPLC-grade methanol, acetonitrile, and n-hexane were acquired from Honeywell (Düsseldorf, Germany).

For spectrophotometric analyses, Merck (Darmstadt, Germany) provided the Folin–Ciocalteu reagent. Chlorogenic acid (5-caffeoylquinic acid), (±)-catechin hydrate, iron (II) sulfate heptahydrate, 2,4,6-tris(2-pyridyl)-s-triazine (TPTZ), 2,2-diphenyl-1-picrylhydrazyl (DPPH), (±)-6-hydroxy-2,5,7,8-tetramethylchromane-2-carboxylic acid (Trolox), and iron (III) chloride hexahydrate were all supplied by Sigma-Aldrich (St. Louis, MO, USA).

For cellular assays, minimum essential medium (MEM), HEPES, trypsin–EDTA, antibiotic/antimycotic solution, NADH, SRB, sodium pyruvate, and trichloroaceric acid were all purchased from Sigma (St. Louis, MO, USA); fetal calf serum was supplied by Invitrogen Corporation (Carlsbad, CA, USA); Triton X-100 was purchased from Merck (Darmstadt, Germany); [1,2-^3^H(N)]-deoxy-D-glucose (^3^H-DG; 60 mCi/mmol) and ^14^C-D-fructose (^14^C-FRU; 250–360 mCi/mmol) were purchased from American Radiolabeled Chemicals (St. Louis, MO, USA).

All other reagents were of analytical grade. Ultrapure water was obtained from a Direct-Pure UP Ultrapure & RO Lab Water System (RephiLe Bioscience Ltd., Boston, MA, USA).

### 2.2. Sample and Sample Preparation

Dried coffee pulp from *Coffea arabica* (caturra vermelha variety) was kindly provided by a local Azorean producer (São Miguel, Azores, Portugal; 37°48’24.4” N 25°37’29.5” W), corresponding to the 2020 harvest (June). Coffee pulp was collected right after harvesting the fruits using a de-pulper (initial step of the wet processing method). It was then carefully spread in a thin layer on a net, in the open air, inside a greenhouse to be protected from direct sunlight and climatic conditions, being frequently turned and moved to dry evenly, until a moisture content lower than 10% was obtained. After being dried, the sample (~2–3 kg) was shipped to the laboratory. There, the ground coffee pulp was stored under vacuum and kept protected from light at room temperature, till analyses.

### 2.3. Nutritional Composition

Ash (AOAC 923.03), crude protein (AOAC 984.13), total fat (AOAC 991.36), and total and insoluble dietary fiber (AOAC 985.29 and 991.42, respectively) were determined using standard normalized methods [16]. Soluble fiber and available carbohydrate contents were estimated by calculation.

### 2.4. Mineral Composition

Sample mineralization was performed in an ETHOS™ EASY microwave oven (Milestone, Sorisole, Italy) equipped with an SK-15 EasyTEMP high-pressure rotor [17,18]. Briefly, 400 mg of sample were mixed with 9 mL HNO_3_ (69% *w*/*w*). After pre-digestion, 1 mL H_2_O_2_ (30% *w*/*w*) was added. The vessels were then tightly closed and placed in a microwave oven (program: temperature increase in 20 min up to 210 °C; 15 min at 210 °C). After cooling down, 0.5 mL of HCl (>30% *w*/*w*) was diluted with ultrapure water. A digestion blank (with reagents only and no sample) was also prepared. To ensure the quality of the digestion process, 400 mg of three different reference materials—BCR-679 (white cabbage), ERM-BB422 (fish muscle), and ERM-BC382 (wheat flour)—were digested under the same conditions as the sample.

For the elemental analysis of the digested sample, an ICP-MS iCAP™ Q (Thermo Fisher Scientific, Bremen, Germany) containing a Meinhard^®^ TQ+ quartz concentric nebulizer (Golden, CO, USA), a high-purity quartz cyclonic nebulization chamber, and a detachable quartz torch with a 2.5 mm internal diameter injector, and an interface of two nickel cones (sampler and skimmer), was used. High-purity argon (99.9997%; Gasin, Leça da Palmeira, Portugal) was used both as nebulization gas and for plasma formation. The operating conditions were as follows: 1.14 L/min of nebulization gas; 0.79 L/min of auxiliary gas; 13.9 L/min of plasma gas; 1550 W of radiofrequency power; and a dwell time ranging from 1 to 50 ms. The internal standard was prepared by proper dilution of single-element Ga and Rh solutions and a multi-element solution (Internal Standard Mix 1–SCP-IS7).

Calibration curves were prepared by properly diluting the stock standard solutions in the following ranges: Mn, Co, Ni, Cu, Zn, As, Se, Rb, Sr, Mo, Cd, Cs, Ba, and Pb (0.5–50 µg/L); Fe (100–1000 µg/L); Mg and Na (100–10,000 µg/L); K, Ca, and P (1000–50,000 µg/L); Hg (1–10 µg/L). The isotopes ^23^Na, ^25^Mg, ^31^P, ^39^K, ^43^Ca, ^55^Mn, ^57^Fe, ^59^Co, ^60^Ni, ^65^Cu, ^66^Zn, ^75^As, ^82^Se, ^87^Rb, ^88^Sr, ^98^Mo, ^111^Cd, ^133^Cs, ^137^Ba, ^202^Hg, ^206^Pb, ^207^Pb, and ^208^Pb were analyzed for sample elemental content, and the isotopes ^6^Li, ^45^Sc, ^71^Ga, ^90^Y, ^103^Rh, ^115^In, and ^209^Bi were monitored as internal standards. The reference materials were analyzed at the beginning and end of the analytical series.

### 2.5. Amino Acid Profile

For the determination of total amino acids, the method outlined by Machado et al. [19] was followed. Very briefly, the sample (~150 mg) was mixed with 6M HCl to undergo an acid hydrolysis (110 °C, 24 h). In parallel, alkaline hydrolysis using 3 mL KOH (4 M) for 6 h was conducted specifically to determine tryptophan content. The hydrolyzed samples were then centrifuged, and a supernatant aliquot (50 μL) was collected and neutralized.

For free amino acids, the protocol described by Machado et al. [19] was also followed. In this case, free amino acids were extracted from the sample (0.5 g) with deionized water (10 mL) by magnetic stirring (40 °C, 30 min), followed by re-extraction (5 mL; 40 °C, 15 min).

The internal standard (norvaline, 2 mg/L) was added to hydrolysates/extracts prior to online automatic derivatization, as described by Machado et al. [19].

Total and free amino acids were quantified via reversed phase-HPLC using an integrated system from Jasco (Jasco, Tokyo, Japan), composed of an LC-NetII/ADC hardware interface, two PU-980 pumps, an AS-4150 automatic derivatizer/injector, an FP-2020 Plus detector, and a CO-4061 oven. Separation of amino acids was carried out on a ZORBAX Eclipse Plus C_18_ column (4.6 × 250 mm, 5 μm; Agilent Technologies, Santa Clara, CA, USA) at 50 °C, using the same elution conditions as those described by Machado et al. [19].

### 2.6. Vitamin E Profile Analysis

Vitamin E was determined as described by Alves et al. [20]. Very briefly, 150 mg of sample was mixed for 30 min with BHT (75 µL, 0.1%), tocol (50 µL, 0.1 mg/mL) and absolute ethanol (1 mL). Then, n-hexane (4 mL) was added, and another vortexing cycle of 30 min was employed. Subsequently, 2 mL of NaCl (1%, *w*/*v*) were added. After vortexing, the organic phase was collected, and the residue was re-extracted with n-hexane. The organic phases were mixed and anhydrous Na_2_SO_4_ was used to ensure that no water was left in the extract. The final solution was concentrated under a N_2_ stream and analyzed using an HPLC system (Jasco, Tokyo, Japan) equipped with an autosampler (AS-4050), a pump (PV-4180), a Supelcosil^TM^ LC-SI column (7.5 cm × 3 mm, 3 µm, Supelco, Bellefonte, PA, USA), a diode-array detector (MD-4015), and a fluorescence detector (FP-4025). The system was operated according to the same conditions described by Alves et al. [20].

### 2.7. Fatty Acid Profile Analysis

The same lipid extraction protocol described in Section 2.5 Vitamin E profile analysis was employed. Fatty acids were then derivatized into methyl esters, according to ISO 12966-2: 2011 [21], using a procedure based on both transesterification and methylation of the free fatty acids. The resulting fatty acid methyl esters (FAMEs) were then analyzed in a GC-FID system (Shimadzu, Tokyo, Japan), equipped with an AOC-20i autosampler, a split/splitless auto-injector (250 °C), and a flame ionization detector. FAME separation was achieved on a CP-Sil 88 silica capillary column (50 m × 0.25 mm, 0.2 µm) from Varian (Middelburg, The Netherlands). The carrier gas was helium (3.0 mL/min), and the temperature program used was 120 °C, 5 min; 2 °C/min till 160 °C; 160 °C, 2 min; 2 °C/min till 220; 220 °C for 10 min. FAMEs were identified by comparing the respective retention times with those of the Supelco 37 Component FAME Mix.

### 2.8. Free Sugar Determination

To extract free sugars, the sample (200 mg) was mixed with deionized water (10 mL) for 20 min. The samples were then centrifuged, and the supernatant was analyzed on an HPLC system (Jasco, Tokyo, Japan) equipped with an autosampler (AS-4050), a pump (PU-4180), a column oven (CO-4061), and a low-temperature evaporative light scattering detector (LT-ELSD Sedex 80, Sedere, Alfortville, France). Fructose, glucose, and sucrose were quantified after chromatographic separation on a Shodex column (Asahipak NH2P-50 4E, 4.6 mm ID × 250 mm) at 30 °C, using water/acetonitrile (1:3) as eluent (1 mL/min) for 20 min [22].

### 2.9. Antioxidant Profile

To obtain antioxidants, 100 mg of sample were extracted with 40 mL water/ethanol (1:1). The extractions were carried out for 1 h at 40 °C with constant agitation. The obtained extracts were filtered and stored at −20 °C for the following analyses.

The ferric-reducing antioxidant power was determined according to Rufino et al. [23] with minor modifications. The extract (35 µL) was mixed with 265 µL of FRAP reagent [23] at 37 °C for 30 min. Absorbance was measured at 595 nm. A calibration curve was prepared with ferrous sulfate (50–600 µmol/L). The DPPH^●^ scavenging activity assay was carried out according to Silveira et al. [24] with minor adjustments. In brief, 30 µL of extract were mixed with 270 µL of an ethanolic DPPH^●^ solution (6·10^−5^ M) at room temperature, protected from light, for 20 min. Absorbance was read at 525 nm. A calibration curve was prepared with Trolox (5–150 mg/L).

Total phenolic content was estimated according to Zhang et al. [25] with some minor alterations. The extract (30 µL) was mixed with 150 µL of Folin–Ciocalteu reagent (1:10) and 120 µL of sodium carbonate (7.5% *w/v*) at 45 °C for 15 min, followed by 30 min at room temperature. Absorbance was measured at 765. Chlorogenic acid (5–160 mg/L) was used to prepare the calibration curve. In turn, total flavonoid content was estimated according to Zou et al. [26] using minor adjustments. The extract (1 mL) was diluted with water (1:5) and mixed with 300 µL of 5% (*w/v*) sodium nitrite. After 5 min, 300 µL of 10% aluminium chloride was added. After one more minute, 2 mL of 1 M sodium hydroxide and 2.5 mL of water were also added and the absorbance was read at 510 nm. Catechin (5–100 mg/L) was used to prepare the calibration curve.

### 2.10. Chlorogenic Acids Profile and Caffeine Content by HPLC

The extracts prepared in Section 2.9. Antioxidant profile were further analyzed to quantify chlorogenic acids and caffeine. An HPLC integrated system from Jasco (Tokyo, Japan) equipped with an automatic sampler (AS-2057 Plus), a pump (PU-2089 Plus), a column oven (CO-2060 Plus, 28 °C), a Zorbax-SB-C18 (5 µm, 250 mm × 4.6; Agilent Technologies, Santa Clara, CA, USA), and a diode-array detector (MD-2018 Plus) was used for analysis, using exactly the same conditions as those described by Machado et al. [27].

### 2.11. Antidiabetic Potential

The Caco-2 cell line (human colorectal adenocarcinoma; passage numbers 14–39) was obtained from ATCC (Manassas, VA, USA). The cells were grown in MEM (containing glucose and supplemented with fetal calf serum, HEPES, and antibiotics), sub-cultured, and prepared, as described by Peixoto et al. [28].

The extracts were prepared as described in Section 2.9. Antioxidant profile. Ethanol was then evaporated under a N_2_ stream and the wet residue was freeze-dried. An amount (~10 mg) of this freeze-dried powder was dissolved in distilled water (100 mg/mL). For each of the following experiments, decreasing concentrations (2, 1, and 0.5 mg/mL) were prepared. Cells were exposed to those concentrations for 24 h in FCS-free culture medium. Distilled water was used as control, and did not influence the measured parameters.

^3^H-DG and ^14^C-FRU uptake studies were performed exactly as described by Peixoto et al. [28]. Cell radioactivity was measured by liquid scintillation counting (LKB Wallac 1209 Rackbeta, Turku, Finland). When tested, the extracts were present for 24 h before the uptake assays (in FCS-free culture medium), and also during preincubation and incubation periods. In controls, the extract was replaced by distilled water.

At the end of 24 h of exposure to the extract, cell viability was also assessed using two distinct methods, the lactate dehydrogenase (LDH) activity assay (which measures the activity of LDH released from cell-membrane-damaged cells) and the sulforhodamine B (SRB) assay (which quantifies total cell proteins) [28]. The protein content of cell monolayers was determined as described by Bradford [29], using human serum albumin as standard.

### 2.12. Statistical Analysis

Data were expressed as average ± standard (*n* = 3) for chemical parameters. For cell experiments, data were presented as average ± standard error of the average (*n* = 9). Student’s *t*-test at *p* < 0.05 was used to evaluate statistical differences between two groups (GraphPad Prism version 7.0 software, San Diego, CA, USA).

## 3. Results and Discussion

Upcycling by-products offers significant opportunities for sustainable advancement across social, environmental, and economic dimensions, contributing to the circular economy requirements within the coffee chain. This approach entails not only prioritizing coffee beans but also recognizing the potential of by-products like cherry pulp, which is frequently discarded despite its valuable properties. The present study aimed to evaluate the chemical composition and the bioactive potential of coffee cherry pulp from São Miguel Island, in the Azores archipelago. Given the distinctive microclimatic conditions of the Azores, we sought to investigate whether the coffee cherry pulp from this region might exhibit specific characteristics that could enhance its bioactive properties.

Table 1 shows the proximate composition (in dry weight) of the dried coffee pulp analyzed in this study, which is essential for determining its potential as a food ingredient. The results showed a diverse and rich nutritional profile, with a high dietary fiber content (52%). The results also showed that the coffee pulp has substantial amounts of total minerals (11%) and total protein (10%), together with a low-fat content (<2%). These results are in the same range as those reported by authors who studied coffee pulp from different geographical origins [27,30,31,32], although some small differences can be noticed. In Table 1, it is possible to compare the nutritional composition of coffee pulps from different species (arabica and robusta) and geographical origins (data extracted from the literature [27,30,31,32]). The results are all expressed as percentage of dry weight (% dw), enabling a cross-comparison of the nutrient levels.

### 3.1. Carbohydrate Profile

Total dietary fiber varied slightly among the samples, with the highest content observed in robusta pulp from Vietnam (54%) and the lowest in Colombian arabica pulp (46%). The similarity between the total fiber content of our sample and that from Vietnam (Table 1) suggests a minor influence of the coffee species in this parameter. However, when looking at the different fiber profiles, it seems that pulp from arabica species is significantly richer in soluble fiber (~2-fold). This is particularly relevant as different fiber types have distinct physiological functions, and the fiber profile will directly influence the suitability of coffee pulp for specific dietary and functional applications. For example, insoluble dietary fiber is recognized for supporting bowel regularity, adding bulk to stool, facilitating intestinal transit, and helping to relieve constipation, thereby improving overall digestive health. In turn, soluble fiber can slow glucose absorption and plays an essential role in gut health through its fermentability, providing a substrate for beneficial gut bacteria and supporting immune function. Additionally, soluble fiber has been linked to an improved lipid profile and glycemic control, which can be particularly beneficial for people managing metabolic conditions such as diabetes [33,34].

In what concerns free sugars, Nicaraguan arabica pulp contained 2.6-fold higher fructose contents and lower contents of glucose compared to our sample, which could be due to differences in growing conditions, agricultural practices, post-harvest processing, and particularly the ripeness of the fruit at the moment of harvest [35,36,37]. These differences will certainly influence the sweetness of the fruit.

### 3.2. Mineral Profile

Regarding the ash content, indicative of the total mineral composition, this showed slight variations among the species and regions. In the current study, we found an ash content of 10.6%, which aligned closely with the Colombian sample (10.7%) but exceeded the values found for Vietnamese robusta pulp (6.3%) [32] and Mexican (7.4%) [31] and Nicaraguan arabica (7.6%) [30] pulps (Table 1). This suggests that geographical factors, including the soil mineral content and cultivation methods, can influence the mineral concentration in coffee pulp. To better understand the mineral profile of the coffee pulp considered in this study, our sample was subjected to further analysis. Table 2 depicts the results obtained by ICP-MS, showing that our Azorean coffee pulp contains various macroelements, essential trace elements, and non-essential or potentially toxic elements, each with unique roles and impacts on human health.

Potassium was the most abundant macroelement (42.5 mg/g), followed by calcium (3.55 mg/g), magnesium (1.84 mg/g), and phosphorus (1.46 mg/g). These results are in accordance with those of Hurtado and Abarca [38], who found a similar macroelement profile (although the potassium content in our sample was slightly higher) in arabica coffee pulp from the Typica variety produced in Ecuador (results in dry basis: 3.1% of potassium, 0.46% of calcium, 0.14% of magnesium, and 0.13% of phosphorus [38]).

The same profile was also reported by Gil-Ramirez et al. [30] for Nicaraguan arabica pulp, although lower concentrations were observed in their study: 24.2 mg/g of potassium, 2.4 mg/g of calcium, 1.2 mg/g of phosphorous, and 0.9 mg/g of magnesium (results in dry weight). This lower amount of macroelements reflects the lower ash content also described by the authors [30] in comparison to our Azorean coffee pulp (Table 1). In addition, in our study, we also found sodium at a concentration of 1.00 mg/g (Table 2). These macroelements are physiologically important in dietary sources since they play critical roles in numerous functions within the human body. For example, potassium is crucial for cellular function and supports numerous physiological processes, such as muscle function, glucose metabolism, and blood pressure, while calcium plays an essential role in blood clotting, muscle contraction, nerve transmission, and the formation of bones and teeth. Magnesium, in turn, is involved in essential enzymatic reactions, including the activation of amino acids, DNA synthesis, and neurotransmission, supporting also the immune function; and sodium works alongside potassium to regulate the distribution of body water and blood pressure, being essential for maintaining acid–base balance and facilitating the transmission of nerve impulses [39]. Meanwhile, phosphorus primarily serves as the main storage form of metabolic energy and acts as a co-factor for various enzymes [39]. In addition to these five macroelements, we also identified essential trace elements in Azoren coffee pulp, such as iron (27 µg/g), copper (13.4 µg/g), zinc (9.3 µg/g), and manganese (8.6 µg/g), followed by molybdenum, selenium, and cobalt in the ng/g range (Table 2), which collectively offer numerous health benefits to human metabolism [39]. Contrary to what was observed for macroelements, for microelements, Gil-Ramirez et al. [30] reported significantly higher values compared to those found in this study: iron (77 µg/g), manganese (62 µg/g), cooper (18 µg/g), and zinc (17 µg/g). The authors also reported the presence of boron and silicon. These differences highly suggest that environmental factors, such as soil composition, climate, and agricultural practices, have a significant impact on the microelement composition of coffee pulp.

Finally, in terms of non-essential and toxic trace elements, several were found in the Azorean coffee pulp (Table 2), with values ranging from 9 ng/g (cadmium) to 135 µg/g (rubidium). Although we reported the presence of more toxic elements (Table 2) in comparison to the study of Gil-Ramirez et al. [30], the Azorean coffee pulp seems to have a lower level of contamination regarding some elements (based on the concentrations found). For example, in the Nicaraguan pulp, Gil-Ramirez et al. [30] found 25 ng/g of cadmium, 1.05 µg/g of nickel, and 0.18 µg/g of arsenic. In turn, lead was absent, while we found it in our samples (15.8 ng/g). Despite these differences, the concentrations of these elements in coffee pulp samples remain generally low. Indeed, the European Food Safety Authority (EFSA) technical report [6] on the notification of dried cherry pulp as a traditional food from a third country provides data on the heavy metal content of six batches of dried coffee cherry pulp (lead: 18–123 ng/g, cadmium: <5–20 ng/g; arsenic: <10–40 ng/g; mercury: <10 ng/g; nickel: <0.05–0.45 µg/g), values that are within the same range or slightly higher than ours (Table 2), which were considered safe and not concerning for market placement.

### 3.3. Protein Profile

**Figure 1:**
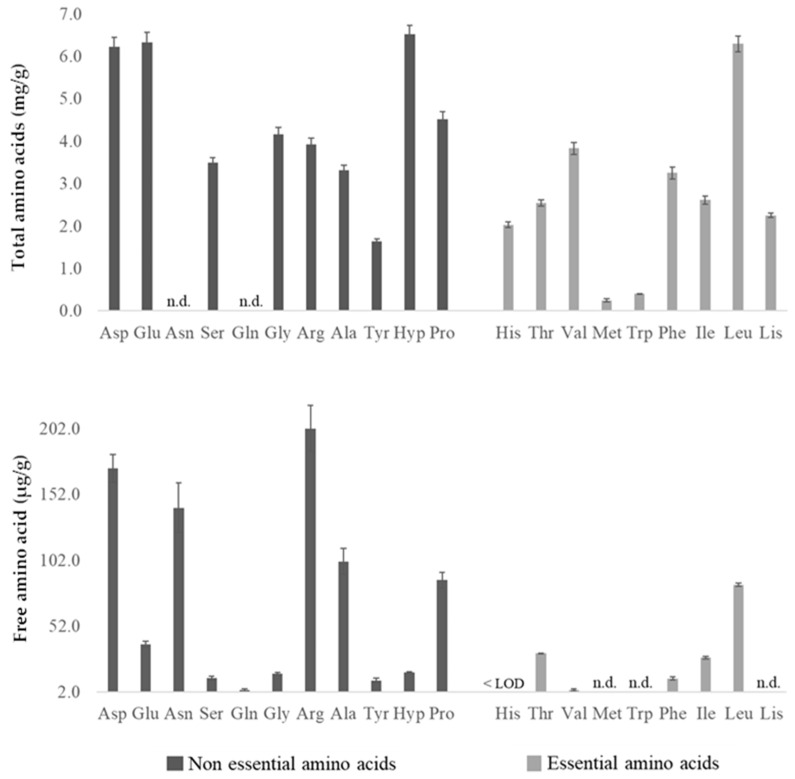
Total and free amino acid profiles of Azorean coffee pulp. The results are expressed as mean ± standard deviation (*n* = 3), in dry weight. n.d., not detected (For total amino acids, conversion of asparagine and glutamine into aspartic acid and glutamic acid occurs during acid hydrolysis, respectively).

The crude protein content was relatively consistent across all samples, averaging around 10%, with the highest value in the Mexican arabica pulp (10.85%) [31] and the lowest in Vietnamese robusta pulp (9.52%) [32]. These values indicate that this by-product can be an interesting and alternative source of protein. To better understand the composition and the quality of the protein fraction, we analyzed the total (mg/g) and free (µg/g) amino acid profiles of the Azorean coffee pulp (Figure 1). The analysis of these profiles gives valuable and additional information to comprehend the nutritional value and the functional properties of the sample, as each amino acid plays a distinct role in physiological processes [40].

The total amino acid content of the Azorean coffee pulp was 63.5 mg/g. This value is significantly lower than that described for crude protein in Table 1. Some hypotheses can explain this discrepancy. Although the Kjeldahl method is a classic and widely accepted approach for determining total nitrogen, which is then converted to an estimated protein content using an established factor, the determined total nitrogen encompasses all nitrogenous compounds, not just proteins. Indeed, non-protein nitrogen sources, such as nitrates or alkaloids like caffeine or related compounds, will raise the estimated crude protein values. In contrast, HPLC-FLD allows the direct measurement of amino acids, avoiding interference from non-protein nitrogen compounds. However, this chromatographic analysis requires hydrolysis to break down proteins into individual amino acids, which can lead to some degradation or loss of the most sensitive amino acids (e.g., methionine, serine), contributing to the underestimation of total amino acid content. In addition, in some cases, peptide bonds may only be partially broken down, depending on their type and structural characteristics. For example, those involving isoleucine and valine, such as isoleucine/isoleucine, valine/valine, valine/isoleucine, and isoleucine/valine, are not always fully hydrolyzed and may remain partially intact [19,41].

As depicted in Figure 1, the most abundant amino acids in the Azorean coffee pulp were hydroxyproline (6.5 mg/g), leucine (6.3 mg/g), glutamic acid (6.3 mg/g), and aspartic acid (6.2 mg/g). It is important to emphasize that these two last values also take into account the conversion of asparagine and glutamine into aspartic acid and glutamic acid, respectively, under the acidic conditions necessary for hydrolysis. Nonetheless, this deamination process, resulting in the corresponding carboxylic acids, does not impact the quantification, as the molecular weights within each amine/acid pair are almost identical [41]. Other amino acids found in high amounts were proline (4.5 mg/g), glycine (4.2 mg/g), arginine (3.9 mg/g), and valine (3.8 mg/g). All essential amino acids were detected (histidine, threonine, valine, methionine, tryptophan, phenylalanine, isoleucine, leucine, and lysine), in an amount that represents about 37% of the total amino acids (23.4 mg/g); however, the relatively low amount of some of them, in particular methionine and tryptophan, impairs protein quality. To enhance its nutritional value, especially for applications such as the production of coffee pulp-derived flour, it would be advisable to complement its consumption with other protein sources rich in methionine and tryptophan. For example, legumes such as chickpeas and lentils are particularly rich in methionine [42], while the cereal grains spelt and oat are rich in tryptophan [43]. These combinations are perfectly suitable and could effectively address the deficiencies in methionine and tryptophan found in coffee pulp, significantly enhancing the overall protein quality. Despite that limitation, coffee pulp can be seen as a very good source of amino acids with important physiological roles that can be used for specific food enrichment. For example, glycine, proline, and hydroxyproline account for 57% of the total amino acid composition in collagen, which is crucial for maintaining the integrity and strength of connective tissues (e.g., skin, bones, cartilage, and blood vessels) [44]. In turn, glutamic acid, aspartic acid, arginine, and proline, are key contributors to cognitive functions, while the branched-chain amino acids (leucine, isoleucine, and valine), also present in substantial amounts, have a relevant role in muscle growth [40].

The total amino acid profile obtained in the present study is quite different from that described by Gil-Ramirez et al. [30]. Although the authors described 12.9% protein content in Nicaraguan arabica coffee pulp (higher than that found in this study), they reported a total amino acid content of only 21 mg/g (~2%). This difference could be explained by the fact that different amino acids were quantified in both studies, probably due to standards availability. For example, we quantified hydroxyproline (a major amino acid in Azorean coffee pulp) and tryptophan (performing an extra basic hydrolysis), while they quantified cysteine (a minor amino acid). However, as this does not explain all the differences observed, the method employed to quantify amino acids should also be considered. For example, Gil-Ramirez et al. [30] used a different hydrolysis protocol and did not refer to a neutralization step before storage to avoid eventual amino acid degradation that may occur even when the sample is frozen at −20 °C. In addition, they used a different derivatization procedure as well as a distinct chromatographic separation method, and that could also influence the results. Overall, the amino acid contents reported in the present study are approximately 2- to 4-fold higher than those reported by Gil-Ramirez et al. [30], with some slight variations.

In Table 3, it is also possible to observe the free amino acid profile of Azorean coffee pulp. Free amino acids, which are not bound in protein structures, are available for rapid absorption and metabolism. They are essential to regulate both exocrine and endocrine secretions and influence protein digestion, metabolic processes, and nutrient absorption, while also contributing to the maintenance of the integrity and protective functions of the gastrointestinal mucosa [45]. In addition, their contribution to the sensorial characteristics of food has long been recognized [46]. The total free amino acid content of the sample analyzed in this study was 0.96 mg/g. Arginine (202 µg/g) and aspartic acid (172 µg/g) were the most abundant free amino acids, followed by asparagine (142 µg/g) and alanine (101 µg/g). In terms of flavor, these four amino acids can be responsible for sweetness (alanine), sourness (aspartic acid), sourness or sweetness (asparagine), and bitterness (arginine). Aspartic acid and alanine, together with glutamic acid (38 µg/g), threonine (31 µg/g), glycine (16 µg/g), serine (12.5 µg/g), and glutamine (3.6 µg/g), are also involved in umami taste [46]. In turn, some essential amino acids, such as histidine, lysine, methionine, and tryptophan were not detected in Azorean coffee pulp. This free profile is also different from that reported by Gil-Ramirez et al. [30]. The authors highlighted proline as the main free amino acid, followed by serine, aspartic acid, phenylalanine, and arginine, in this order; they also reported the presence of free histidine, lysine, and methionine, although in low contents. Several factors could have influenced the free amino acid profile of the samples analyzed in the different studies, leading to these variations, such as the growing conditions, the maturity at harvest, and post-harvest handling. In fact, even for the coffee bean, there are several studies that reveal the influence of these same factors on the free amino acid profile [47,48,49].

### 3.4. Lipid Profile

Although coffee pulp is a good source of amino acids and minerals, it is very low in fat. However, some differences can be highlighted among samples collected from distinct geographical origins and species (Table 1). For example, the Nicaraguan arabica pulp contained the highest total fat content (2.8%) [30], while the Vietnamese robusta pulp had the lowest (1.2%) [31].

Despite this low fat content, coffee pulp seems to be a good source of vitamin E, which is a liposoluble antioxidant comprised of eight naturally occurring compounds that share similar structures (vitamers). Their structures contain a chromanol ring with different methyl group arrangements (α-, β-, γ-, and δ-), and a 16-carbon phytyl side chain fully saturated in tocopherols and unsaturated in tocotrienols [20]. The eight forms show different levels of biological activity, being α-tocopherol the most active due to its selective recognition by the α-tocopherol transfer protein that plays a crucial role in maintaining plasma α-tocopherol levels [50]. Although vitamin E’s biological effect is mostly derived from its antioxidant properties, by protecting cells from peroxidation and contributing to membrane integrity, it is also involved in the primary intracellular defense system and has been associated with the prevention of several diseases [51].

In a previous work published by our group [20], we found that from the eight possible vitamers, coffee beans contained only α- and β-tocopherols, with β-tocopherol present in approximately twice the amount of α-tocopherol in arabica coffee beans (both raw and roasted). In the case of the Azorean arabica pulp (total vitamin E: 72.67 ± 1.01 µg/g dw), besides α- and β-tocopherols, (51.18 ± 1.26 and 2.12 ± 0.07 µg/g dw, respectively) we also identified γ-tocopherol (19.37 ± 0.77 µg/g dw). However, in this case, α-tocopherol was the major vitamer (approximately 2.6- and 25-fold higher than γ- and β-tocopherols, respectively), being this a more desirable profile based on α-tocopherol higher biological activity. These results are in accordance with those reported by Tavares et al. [52] for coffee husks (the main by-product obtained from the dry post-harvest coffee processing), which consists of dried skin, pulp, mucilage, and parchment. The authors found a vitamin E profile similar to that reported above for our sample, with α-tocopherol being the major vitamer (3.7–7.1 mg/100 g), followed by γ-tocopherol (1.2–2.1 mg/100 g) and β-tocopherol (0.4–0.9 mg/100 g) [52].

In terms of the fatty acid profile of Azorean coffee pulp (Table 3), palmitic acid was the major fatty acid found (41%), contributing significantly to the total saturated fatty acids (SFAs) content (55%). Consequently, the other SFAs—stearic acid (8.9%), arachidic acid (3.8%), and myristic acid (1.4%)—were present in lower amounts. Although the lipid profile showed this predominance in SFAs, polyunsaturated fatty acids (PUFAs) were also detected in substantial amounts (38%). The essential linoleic and α-linolenic acids were the main PUFAs (22 and 16%, respectively), resulting in an n6/n3 ratio of 1.4. This falls perfectly within the recommended range for a balanced intake of omega-6 and omega-3 fatty acids, since it has long been recognized that a low n6/n3 ratio (ideally near 1) is crucial for suppressing inflammation and reducing the risk of developing cancer, cardiovascular diseases, and autoimmune disorders [53]. Finally, two monounsaturated fatty acids (MUFAs) were also detected, but not in significant amounts (oleic acid: 5%; palmitoleic acid: 1.5%) when compared to the above-mentioned fatty acids.

The fatty acid profile found in this study for the Azorean coffee pulp is similar to that reported by Rios et al. [54] for dried arabica coffee pulp (Tabi variety), obtained through wet processing in Colombia, suggesting that the variety and/or geographical origin do not have a major influence the lipid profile of the fruits. In their study, the authors reported 47% SFAs, 40% PUFAs, and 12% MUFAs, with the major fatty acids being palmitic acid (36%), linoleic acid (22%), and α-linolenic acid (17%) [54]—very similar values to those presented in Table 3 for Azorean coffee pulp.

### 3.5. Antioxidant and Phytochemical Profile

Azorean coffee pulp presented a high antioxidant capacity (Table 4), evidenced by its ferric-reducing antioxidant power (487.5 µmol FSE/g) and DPPH^●^ scavenging activity (21.5 mg TE/g). These two methods were used in this study because they present complementary mechanisms of action. The FRAP assay involves the reduction of the complex formed between Fe^3+^ and 2,4,6-tripyridyl-s-triazine (TPTZ), which turns blue and is then quantified spectrophotometrically. This method is effective for compounds with redox potentials below 0.7 V, corresponding to the redox potential of Fe^3+^-TPTZ, being a useful in vitro indicator of a compound’s capacity to maintain redox balance in biological systems. In this way, the FRAP assay operates exclusively through an electron transfer mechanism, which means that it cannot detect antioxidants that neutralize radicals via hydrogen atom transfer. In contrast, the DPPH^●^ scavenging assay evaluates antioxidants capable of neutralizing the DPPH^●^ radical either by electron transfer or hydrogen atom transfer. This method measures the antioxidant-reducing capacity by observing the decrease in absorbance of the DPPH^●^ solution, reflecting the reduction or quenching of the radical [55].

The FRAP value found in the present study was slightly lower than those reported by Machado et al. [27] for Colombian arabica pulp (8.58 g FSE/100 g or, after conversion, 565 µmol FSE/g) using a similar protocol, while the DPPH^●^ scavenging activity in our sample was higher (7.7 mg TE/g in the Colombian one). These differences suggest differences in phytochemical composition that could result, as mentioned above, from growing, ripening, or post-harvest processing conditions. To explore that, total flavonoids, total phenolics, chlorogenic acid profile, and caffeine contents were also studied. We found differences in the phenolic profile that could influence the results in the antioxidant assays. In the present study, Azorean coffee pulp presented higher values for total flavonoid (21.7 mg CE/g) and total phenolic contents (45.9 mg CGAE/g) than those described for Colombian coffee pulp (12.3 mg CE/g and 23.7 mg CGAE/g) [27]. In addition, our Azorean sample was also richer in chlorogenic acids (5-caffeoylquinic acid: 8.0 mg/g vs. 2.2 mg/g for Colombian coffee pulp [28]; 4-caffeoylquinic acid: 0.8 mg/g vs. 0.1 mg/g; 3-caffeoylquinic acid: 0.40 vs. 0.06 mg/g, respectively). Notwithstanding, although total flavonoids and other phenolic compounds have an established role as primary contributors to the antioxidant capacity of coffee pulp, other components (such as vitamin E, described in Section 3.4) also play a significant role in this property. In addition, our sample was also slightly richer in caffeine: 9.8 mg/g, against 0.85 mg/g reported by Machado et al. [27] for Colombian pulp.

This richness in phytochemical compounds, such as caffeine and chlorogenic acids, suggests potential beneficial physiological effects when coffee pulp is consumed. By comparison with coffee beans, particularly arabica beans, that contain caffeine in a similar range (~1%) [56], we can infer similar physiological actions. Caffeine is well-documented for its psychoactive effects, including improvements in cognitive and psychomotor performance, with minimal effective doses around 12.5–50 mg [57,58]. Based on the caffeine content in coffee pulp, approximately 1–5 g of coffee pulp flour would be required to reach this threshold. Based on this, coffee pulp could be suggested as a natural source of caffeine to enrich specific foods aimed at enhancing mental and physical performance. However, it is important to consider potential limitations to its consumption. Specific populations, such as children, pregnant or breastfeeding women, and those sensitive to caffeine may avoid or limit intake. Furthermore, excessive consumption should be avoided, as consuming more than 20 g of coffee pulp flour could exceed the safe caffeine limit for a single dose (200 mg) established by the EFSA [59]. In addition, the cumulative caffeine intake from all sources, including coffee beverages, food supplements, energy drinks, medications, and other natural caffeine-containing foods, should also be taken into account to prevent exceeding the recommended daily limits of caffeine ingestion (400 mg) [59].

Chlorogenic acids (esters of hydroxycinnamic acids with quinic acid) have also been associated with several health benefits, including a significant reduction in the risk of developing type II diabetes. Indeed, in recent years, their role in the regulation of sugar and lipid metabolism has been studied, with reports of relevant antioxidant, antidiabetic, and anti-inflammatory effects [60].

### 3.6. Antidiabetic Potential

Although some studies have already reported antidiabetic effects of coffee pulp, namely an inhibitory effect on α-amylase enzyme activity in vitro [61] and the ability to decrease plasma glucose levels and insulin resistance in diabetic rats [12], as far as we know, there are no studies reporting the effects of coffee pulp on intestinal glucose and fructose uptake. Considering that the intestine is an organ of primary importance for the absorption of dietary sugar, the inhibition of this process might be a useful therapeutic strategy to modulate postprandial glycemic control, sugar metabolism, and metabolic health. In this context, radiolabeled glucose (^3^H-deoxyglucose, ^3^H-DG) and fructose (^14^C-fructose, ^14^C-FRU) were used as tracers to study the transport dynamics in Caco-2 cells.

**Figure 2:**
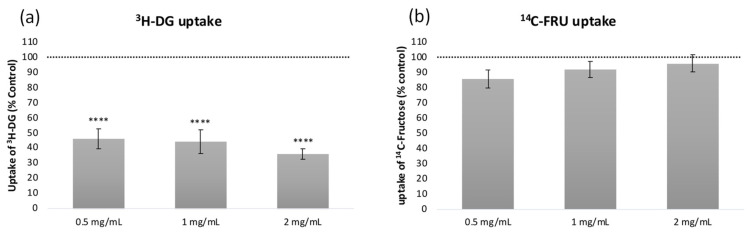
Effect of coffee pulp extracts on (**a**) ^3^H-DG and (**b**) ^14^C-FRU uptake by Caco-2 cells in comparison with control (100%, horizontal dash line). The results are expressed as average ± SEM (*n* = 9); ****, *p* < 0.0001 significantly different from control by Student’s *t*-test.

The effects of Azorean coffee pulp on the glucose (^3^H-DG) and fructose (^14^C-FRU) uptake by Caco-2 cells are depicted in Figure 2.

As can be observed, the Azorean coffee pulp extract was able to significantly inhibit ^3^H-DG uptake at all concentrations tested, while no significant effects (*p* > 0.05) were found on ^14^C-FRU uptake (Figure 2). Of note, the reductions in ^3^H-DG uptake caused by the extract were very expressive (from 54 to 64%) and concentration-dependent.

In the intestine, the absorption of glucose in the apical membrane of small intestinal cells is mediated by the sodium-dependent glucose co-transporter (SGLT1) and by the facilitative glucose transporter 2 (GLUT2), while the absorption of fructose is mediated by the facilitative glucose transporter 5 (GLUT5) and by GLUT2 [62]. Considering the differential effects of the extract on ^3^H-DG and ^14^C-FRU uptake, it might be suggested that SGLT1 is the main target of the extract’s inhibitory effect.

These results are quite encouraging and might be intimately related to the richness of coffee pulp extract in caffeine and chlorogenic acids CGA (Table 4). In fact, in a previous study conducted in our laboratory with extracts of coffee silverskin (a coffee by-product obtained during bean roasting), we found a synergistic effect between 5-caffeoylquinic acid and caffeine in inhibiting both ^3^H-DG and ^14^C-FRU uptake. Regardless, the extracts presented even higher reductions compared to the mixture of both standards at the concentrations present in the extracts [28], highlighting the potential contribution of other compounds besides 5-caffeoylquinic acid and caffeine to the effects found.

**Figure 3:**
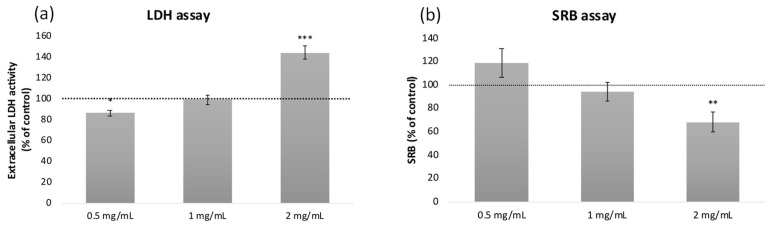
Effect of coffee pulp extracts on (**a**) Caco-2 cell viability (extracellular LDH activity), and (**b**) culture mass (SRB assay), in comparison with control (100%, horizontal dash line). The results are expressed as average ± SEM (*n* = 9). *, *p* < 0.05; **, *p* < 0.01; ***, *p* < 0.001 significantly different from control by Student’s *t*-test.

To confirm that the effects found on ^3^H-DG and ^14^C-FRU uptake were not related to a cytotoxic effect of the coffee pulp extract on Caco-2 cells, LDH and SRB assays were further performed. Among the concentrations tested, the coffee pulp extract presented a significant cytotoxic effect at 2 mg/mL, observable with both the LDH (Figure 3a) and SRB (Figure 3b) assays. However, the extract presented a protective effect in the LDH test at the lowest concentration tested (0.5 mg/mL) (Figure 2a). Therefore, up to 1 mg/mL, the extract is able to decrease ^3^H-DG uptake without interfering with cell viability, which is important in the context of further studies using this coffee pulp extract.

## 4. Conclusions

In this work, a comprehensive analysis of the chemical composition and bioactive potential of coffee cherry pulp from S. Miguel, Azores (Portugal) was carried out. Azorean coffee pulp proved to be a valuable natural resource, particularly from a circular economy perspective, as it constitutes a commonly discarded by-product that can be upcycled into functional ingredients or nutraceuticals.

Chemical analyses revealed that Azorean coffee pulp is a good source of fiber (predominantly insoluble), minerals (mainly K, Ca, and Mg), and protein (with hydroxyproline, aspartic acid, glutamic acid, and leucine being the major amino acids). Although its fat content is low and mostly saturated, the pulp also provides considerable amounts of polyunsaturated fatty acids with a favorable n6/n3 ratio (1.40), as well as vitamin E (mainly α-tocopherol). Its antioxidant capacity can be partially attributed to chlorogenic acids (3-, 4-, and 5-caffeoylquinic acids), and its caffeine content is comparable to that found in arabica coffee beans. In addition to this rich phytochemical profile, a decrease in glucose uptake (but not in fructose uptake) was observed in Caco-2 cells, suggesting selective inhibition of the SGLT1 transporter and a potential antidiabetic effect. These results show that Azorean coffee pulp has potential as a sustainable and bioactive ingredient for incorporation into innovative functional foods or dietary supplements with health-promoting properties.

From a sustainability point of view, upcycling coffee pulp can offer several advantages, since coffee producers can reduce the environmental impact associated with pulp disposal, addressing waste management challenges, while generating additional revenues. Overall, coffee pulp upcycling can contribute to a more resilient and resource-efficient coffee supply chain, promoting local economic development and aligning with global sustainability goals.

## Figures and Tables

**Table 1:** Nutritional composition of coffee pulp (% dry weight) from different species/varieties and geographical origins.

	Current Study *	Machado et al. [27]	Gil-Ramirez et al. [30]	Ameca et al. [31]	Phuong et al. [32]
Coffee species (variety)	Arabica (caturra vermelha)	Arabica	Arabica (caturra)	Arabica	Robusta
Geographical origin	Azores, Portugal	Colombia	Nicaragua	Mexico	Vietnam
Composition:					
Total ash	10.6 ± 0.3	10.7 ± 0.2	7.6 ± 0.1	7.4 ± <0.1	6.3
Crude protein	10.1 ± 0.1	10.2 ± 0.1	12.9 ± 0.0	10.9 ± 0.2	9.5
Total lipids	1.6 ± <0.1	1.7 ± <0.1	2.8 ± 0.2	1.2 ± 0.1	1.2
Total dietary fiber	52.0 ± 0.7	46.1 ± <0.1	49.8 ± 1.7	49.3 ± 2.6	53.9
Insoluble	44.4 ± 0.1	37.0 ± 0.1	38.6 ± 0.5		49.6
Soluble	7.6 ± 0.7	9.1 ± 0.1	11.2 ± 1.2		4.4
Available carbohydrates *	25.6 ± 0.7	31.2 ± 0.3	26.9 ± 3.7		
Free sugars	5.0 ± 0.1				9.2
Frutose	2.9 ± <0.1		7.6 ± 0.3		
Glucose	2.0 ± <0.1		1.6 ± 0.8		
Saccharose	n.d.				
Arabinose			1.6 ± 0.1		

The results within the first column are expressed as mean ± standard deviation (*n* = 3). * Available carbohydrates were estimated by subtracting the ash, protein, fat, and total dietary fiber contents to 100%. n.d., not detected.

**Table 2:** Mineral profile of Azorean coffee pulp.

Macroelements	
K (mg/g)	42.50 ± 1.20
Ca (mg/g)	3.55 ± 0.08
Mg (mg/g)	1.84 ± 0.03
P (mg/g)	1.46 ± 0.04
Na (mg/g)	1.00 ± 0.02
**Essential trace elements**	
Fe (µg/g)	27.20 ± 1.30
Cu (µg/g)	13.50 ± 0.40
Zn (µg/g)	9.26 ± 0.49
Mn (µg/g)	8.63 ± 0.24
Mo (ng/g)	663.03 ± 21.02
Se (ng/g)	64.20 ± 4.80
Co (ng/g)	28.0 ± 1.30
**Non-essential and toxic trace elements**	
Rb (µg/g)	135.22 ± 2.61
Sr (µg/g)	36.67 ± 0.43
Ba (µg/g)	7.05 ± 0.22
Al (µg/g)	6.80 ± 1.05
Ni (µg/g)	0.17 ± 0.01
Cs (ng/g)	76.80 ± 2.20
Li (ng/g)	20.70 ± 2.10
Pb (ng/g)	15.80 ± 1.50
Cd (ng/g)	8.87 ± 0.61
Be (ng/g)	<LOD (2)
As (ng/g)	<LOD (40)
Sb (ng/g)	<LOD (40)
Hg (ng/g)	<LOD (10)
Tl (ng/g)	<LOD (3)

The results are expressed as mean ± standard deviation (*n* = 3), in dry weight. LOD, limit of detection.

**Table 3:** Fatty acids profile of Azorean coffee pulp.

Fatty Acids		
Myristic	C14:0	1.43 ± 0.13
Palmitic	C16:0	40.92 ± 0.28
Palmitoleic	C16:1	1.45 ± 0.10
Stearic	C18:0	8.88 ± 0.29
Oleic	C18:1*n*9*c*	5.33 ± 0.21
Linoleic ^1^	C18:2*n*6*c*	22.30 ± 0.05
Arachidic	C20:0	3.78 ± 0.29
α-Linolenic ^1^	C18:3*n*3	15.91 ± 0.22
n6/n3		1.40 ± 0.02
n9/n6		0.24 ± 0.01
ΣSFA		55.01 ± 0.30
ΣMUFA		6.78 ± 0.21
ΣPUFA		38.21 ± 0.22

The results are expressed as relative % of total fatty acids (mean ± standard deviation, *n* = 3). ^1^ Essential fatty acid. ΣSFA, sum of saturated fatty acids (C14:0 + C16:0 + C18:0 + C20:0); ΣMUFA, sum of monounsaturated fatty acids (C16:1 + C18:1*n*9*c*); ΣPUFA, sum of polyunsaturated fatty acids (C18:2*n*6*c* + C18:3*n*3).

**Table 4:** Antioxidant activity and phytochemical composition of dried Azorean coffee pulp.

Antioxidant Activity	
Ferric reducing antioxidant power (µmol FSE/g dw)	487.47 ± 7.34
DPPH^•^-SA (mg TE/g dw)	21.49 ± 1.62
**Phytochemicals**	
Total flavonoids content (mg CE/g dw)	21.72 ± 0.11
Total phenolics content (mg CGAE/g dw)	45.87 ± 2.00
3-caffeoylquinic acid (mg/g dw)	0.40 ± 0.03
4-caffeoylquinic acid (mg/g dw)	0.83 ± 0.13
5-caffeoylquinic acid (mg/g dw)	7.97 ± 0.89
Caffeine (mg/g dw)	9.82 ± 0.58

The results are expressed as mean ± standard deviation (*n* = 9, for spectrophotometric assays; *n* = 3, for HPLC analyses). FSE, ferrous sulfate equivalents; DPPH^•^-SA, 2,2 diphenyl-1picrylhydrazyl radical scavenging activity; TE, trolox equivalents; CE, catechin equivalents; CGAE, chlorogenic acid equivalents.

## Data Availability

The original contributions presented in this study are included in the article. Further inquiries can be directed to the corresponding author.
